# Governance of responsible research and innovation: A social welfare, psychologically grounded multicriteria decision analysis approach

**DOI:** 10.1016/j.heliyon.2024.e40863

**Published:** 2024-12-04

**Authors:** Harold Paredes-Frigolett, Andreas Pyka, Alexandre Bevilacqua Leoneti, Pablo Nachar-Calderón

**Affiliations:** a Department of Management, School of Management and Economics, Diego Portales University, Av. Santa Clara 797, Huechuraba 8580000, Santiago, Chile; b Institute of Innovation Economics (520i), University of Hohenheim, Wollgrasweg 23, 70599 Stuttgart, Germany; c School of Economics, Administration and Accounting, University of São Paulo, Av. Bandeirantes 3900, Ribeirão Preto 14040-900, Brazil

**Keywords:** Governance of responsible research and innovation, Innovation ecosystems, Multicriteria decision analysis, Social choice theory, Welfare economics

## Abstract

Our article deals with the governance of responsible research and innovation (RRI) and aims to set out a first psychologically grounded decision-theoretic method for the governance of RRI. We approach the governance of RRI as a multicriteria group decision analysis problem of delivering social welfare in an innovation ecosystem. Following such a methodological approach, we develop a psychologically grounded multicriteria group decision analysis method that integrates in its value function the main psychological effects captured in the value function of prospect theory as the main theory of individual decision-making under risk. The method first applies a psychologically motivated multicriteria decision analysis function that measures the welfare delivered to all stakeholders involved in a research and innovation consortium. The method then applies a social welfare function on the welfare measurements of stakeholders to propose a social welfare solution that emerges as an RRI-compliant solution for the consortium. The results are a first psychologically grounded multicriteria group decision analysis method and its first application to the governance of RRI. The implications of our results are theoretical but also practical, as our method contributes not only to the established field of multicriteria decision analysis by setting out new method but also to the field of RRI by delivering a psychologically grounded decision-theoretic method for the governance of RRI.

## Highlights

• We set out a method for governing responsible research and innovation (RRI).
• The method construes RRI as a process of generating social welfare in society.
• The method operationalizes RRI following a multi-stakeholder approach.
• The method uses psychologically grounded individual and social welfare functions.
• The method can be used to generate RRI-compliant technology innovation strategies.

## 1 Introduction

In the last two decades, the European Commission has played a key role in the emergence of responsible research and innovation, RRI hereinafter, as a new field of inquiry. This has been the result of a number of initiatives aimed at developing a framework for the governance of RRI in the European Union. Despite these efforts, however, RRI is still today an emerging field that lacks a widely accepted definition.

The definition of RRI endorsed by the European Commission was proposed by von Schomberg [1], [2], [3] and reads as follows: *“A transparent, interactive process by which societal actors and innovators become mutually responsive with a view to the acceptability, sustainability and societal desirability of the innovation process and its marketable products (to allow a proper embedding of scientific and technological advances in society).”*

More recently, Stilgoe, Owen, and Macnaghten [4] offer the following definition of responsible innovation: *“responsible innovation means taking care of the future through collective stewardship of science and innovation in the present,”* which is a definition that is more aligned with the future-oriented approach to the governance of RRI that we will endorse in this article.

But a more fundamental and relevant problem than the lack of a generally accepted definition of RRI is the lack of an RRI construct as a basis on which to develop a general framework for the governance of RRI.

### 1.1 The RRI construct and the operationalization of RRI governance

Based on a comprehensive body of theoretical and empirical scholarly work on RRI [1], [2], [3], [5], [6], [7], [8], [9], [10], Stilgoe, Owen, and Macnaghten [4] proposed that RRI, as a construct that they left unspecified, should be based on the following dimensions:

1. anticipation;
2. reflexivity;
3. inclusion; and
4. responsiveness.

**Figure 1:**
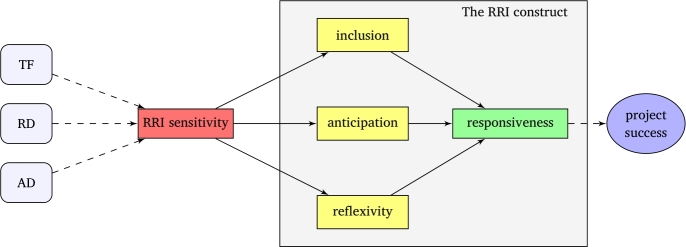
The RRI construct as proposed in [14, p. 127].

Conventional governance models of RRI have traditionally been motivated by risk-based regulation inspired in the precautionary principle [11]. These risk-based approaches, which have led to governance models of RRI based on the precautionary principle, are fundamentally at odds with future-oriented approaches to RRI governance [12]. This departure from risk-based approaches has led to future-oriented approaches to the governance of RRI that draw upon an RRI construct that was yet to be specified. With its focus on responsiveness, and under consideration of the other three dimensions proposed by Stilgoe, Owen, and Macnaghten [4], rather than solely on anticipation [13], a first RRI construct that shall serve as the basis of future-oriented approaches to RRI governance was proposed by Paredes-Frigolett [14, p. 127] and is shown in Fig. 1.

In Fig. 1, “the light blue rectangles with rounded corners represent moderating variables, dashed lines represent the effect of moderating variables, red/green rectangles represent independent/dependent variables, yellow rectangles represent mediating variables, the big gray rectangle represents the RRI construct, and the blue ellipse represents a separate construct called *project success*” [14, p. 127].

As noted by Paredes-Frigolett [14, p. 127], “the independent variable *RRI sensitivity* is moderated by the thematic focus (TF), research direction (RD), and application domain (AD) of a project and the dependent variable *responsiveness* is mediated by the variables *anticipation, reflexivity,* and *inclusion*” [14, p. 127]. This theoretical model postulates further that “the responsiveness of a project acts as moderating variable of another construct called project success” [15, p. 915].

Despite the urgent need to come up with general frameworks for the governance of RRI able to inform and guide RRI in industry and academia, scholarly work published in the Journal of Responsible Innovation, the main outlet for scholarly research in this field, has been dominated by qualitative approaches [16] and the use of methodologies based on multicriteria decision analysis to solve the toughest problem in this field, namely, the governance of RRI, are still underrepresented.

### 1.2 Multicriteria decision analysis approaches to the governance of RRI

The idea of using multicriteria decision-theoretic tools in business ethics dates back to early contributions by Fritzsche [17] and Hosseini and Brenner [18]. Although these initial contributions identified the importance of a research agenda at the intersection of strategy and business ethics to propose models of governance based on decision-theoretic approaches [19], there has been little interest on the part of scholars in the related field of responsible research and innovation to propose such integrative governance frameworks based on decision-theoretic approaches. In one of the most cited articles among RRI scholars, Stilgoe, Owen, and Macnaghten [4, p. 1573] proposed the use of governance tools drawing upon stage-gating decision-making processes to implement responsiveness in a framework for responsible innovation, but they neither elaborated on the theoretical and mathematical foundations for the development of such tools nor described how to implement them.

Recent scholarly work on the governance of RRI has addressed the lack of governance tools by proposing decision-theoretic frameworks for the governance of RRI based on multicriteria decision analysis and by integrating them with agent-based models and simulation for policy engineering [14], [15], [20]. More recently, Paredes-Frigolett, Singer and Pyka set out a framework for ethical research and innovation that embeds RRI within a more general framework of business ethics and proposed a general methodology for implementing this framework using a multicriteria group decision-theoretic approach involving multiple internal and external stakeholders [21]. Despite the work on formal frameworks for RRI governance mentioned above, scholarly research leading to actual tools for RRI governance has remained scanty.

Bellamy, Chilvers, Vaughan, and Lenton [22] were among the first to recognize the need for decision-theoretic models of RRI governance. In their third recommendation for future research, these authors proposed that “multicriteria decision analysis is appropriate to develop decision-aiding tools for the governance of responsible research and innovation in environmentally sensitive areas.” Endorsing such a decision-theoretic approach, Stilgoe, Owen, and Macnaghten [4], on the other hand, were among the first to attempt the development of a framework for RRI governance. In their article, these authors concluded that “*the case* [of the SPICE project analyzed in the context of the framework for RRI governance developed by these authors] *highlights the potential for a framework to inform decision-making in a field with limited governance”* [4, p. 1576].

Recognizing that the governance of RRI is at its core a multicriteria decision analysis problem, Paredes-Frigolett [14] set out a method for classifying the governance of an RRI project based on the four dimensions proposed by Stilgoe, Owen, and Macnaghten [4]. While this was a first step toward the governance of RRI as a decision-making problem involving multiple stakeholders and criteria, the operationalization of a general framework for the governance of RRI is still missing in the RRI literature.

A first operationalization of a framework for ethical research and innovation was proposed by Paredes-Frigolett, Singer, and Pyka [21]. This framework extended current frameworks for RRI governance from two different perspectives. The first one was the integration of frameworks for RRI governance with models of business ethics [23]. The second one was the operationalization of a framework for RRI governance following a multicriteria decision analysis approach.

However, the multicriteria decision analysis method used by this framework did not elaborate on the problem of social choice associated with aggregating and resolving the different preferences of the stakeholders involved and the potential conflicts that may arise in a research and innovation project. These authors merely assume a rather simple plurality rule for aggregating the preferences of stakeholders without considering other aggregations logics [21, p. 34], let alone aggregations logics based on principles of optimizing social welfare in complex innovation ecosystems.

The use of multicriteria decision analysis to solve the governance problem of RRI begs the question of whether or not current multicriteria decision analysis methods are up to this challenging task.

### 1.3 Filling the gap

The case for using multicriteria decision analysis approaches to the governance of RRI is strong for several reasons. Chief among them is the fact that research and innovation consortia lead to the formation of complex innovation ecosystems comprised of heterogeneous internal stakeholders within the consortia and external stakeholders in the innovation ecosystem, each one of them with different profiles. These different profiles manifest themselves in different and often competing vectors of weights for a given set of criteria, which are used to evaluate different alternatives in pursuit of different and often conflicting objectives. But a more fundamental question that arises when considering multicriteria decision analysis approaches to the governance of RRI is not whether or not they can be applied but rather whether or not they need to be extended to serve as effective tools for guiding research and innovation in a responsible manner. In this article, we argue that they do indeed need to be extended for the reasons we describe below.

Research and innovation entail processes that are inherently ridden with strategic uncertainty. This brings the psychology of individual decision-making under risk into the picture and raises the need to replace the value functions of multicriteria decision analysis methods based on expected utility theory [24] and subjective expected utility theory [25] with theories of human decision-making under risk such as prospect theory [26]. This brings to our attention the need to extend conventional multicriteria decision analysis methods by bringing the psychology of individual and group decision-making under risk into their individual and social welfare value functions in order to render them psychologically plausible.

As mentioned, research and innovation consortia entail complex innovation ecosystems comprised of stakeholders pursuing different and often conflicting objectives. The overall objective of RRI governance in such complex innovation ecosystems shifts from the pursuit of value maximization for a reduced set of stakeholders to the pursuit of value optimization across all stakeholders involved in the innovation ecosystem. This calls for the integration of different social welfare functions to implement different aggregation logics as part of the group decision-making processes of the multicriteria decision analysis methods deployed. By following a social welfare approach to the development and implementation of multicriteria decision analysis methods for the governance of RRI we not only benefit from a comprehensive body of scholarly work in the welfare economics [27] and social choice theory [28], [29] but can also achieve a cross-pollination effect by contributing to these more mature fields with new families of social welfare functions.

The article is organized as follows. In Section 2, we present a two-step methodology for the governance of RRI that deploys a multicriteria group decision analysis method allowing research organizations to evaluate and resolve the different governance trade-offs that arise when conducting a research and innovation project. In Section 3, we present our results based on the case of a highly sensitive RRI project and compare our results with other multicriteria decision analysis methods. In Section 4, we present a discussion of our main results. In Section 5, we present our conclusions and ideas for future work.

## 2 Methodology

We now present a two-step methodology that can be used to develop decision-aiding tools for the governance of RRI.

### 2.1 First step: eliciting the welfares of stakeholders in an innovation consortium

The first step of the methodology consists in eliciting the welfares of all stakeholders that are members of an innovation consortium and participate in a research and innovation project. This is accomplished by introducing a psychologically motivated welfare function of alternatives for stakeholders.

Definition 2.1 **The set of alternatives**

Let $A=\{a_{i}\}$, with 1≤i≤m, be the set of alternatives.

Definition 2.2 **The set of criteria**

Let $C=\{c_{j}\}$, with 1≤j≤n, be the set of criteria used to evaluate the alternatives in *A*.

Definition 2.3 **The set of stakeholders**

Let $S=\{s_{k}\}$, with 1≤k≤o, be the set of stakeholders who evaluate each alternative $a_{i}\in A$ using the criteria $c_{j}\in C$.

Definition 2.4 **The vector of normalized weights of stakeholders**

Let ${\overset{\to }{w}}^{k}$ be the vector of normalized weights of stakeholder $s_{k}\in S$, where ${\overset{\to }{w}}^{k}=(w_{j}^{k})$ and $w_{j}^{k}$ is the weight that stakeholder $s_{k}\in S$ attaches to criterion $c_{j}\in C$, with $w_{j}^{k}\in R\in [0,1]$ and 1≤j≤n and 1≤k≤o. The vector of normalized weights of stakeholders is calculated using the ranking order centroid (ROC) method [30].

Remark 1 Proportion-based methods such as the ROC method are more efficient for determining preference weights than methods that rely on pairwise comparisons of criteria, such as the AHP method [31]. This is due to the fact that pairwise comparisons methods must compare all pairs of criteria for all alternatives, which renders these methods less computationally tractable [32] and produces a higher number of ties among criteria, thus leading to vector of weights that are less granular and thus less informative. Due to their higher granularity and computational tractability, proportion-based methods based on ranking orders of criteria such as the ROC method are usually preferred [30].

Definition 2.5 **The matrix of evaluation scores**

Let $E_{mn}$ be the matrix of evaluation scores containing the scores $e_{ij}$ for all 1≤i≤m and 1≤j≤n, where $e_{ij}$ is the value of criterion $c_{j}\in C$ for alternative $a_{i}\in A$, with 1≤i≤m, 1≤j≤n.

Definition 2.6 **The matrix of normalized scores**

Let $N_{mn}$ be the matrix containing the normalized scores $n_{ij}$, where $n_{ij}$ is defined as follows:(1)
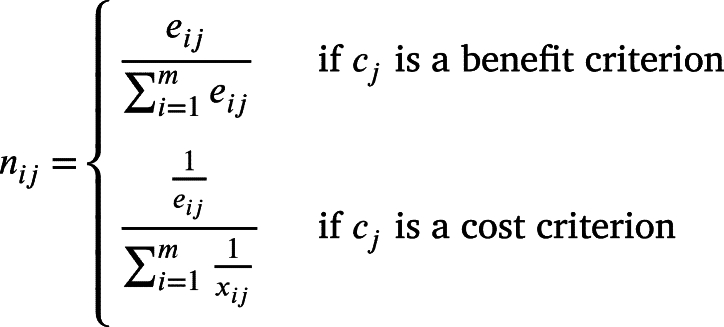
 with $n_{ij}\in R\in [0,1]$ for all 1≤i≤m and 1≤j≤n.

We now introduce a psychologically grounded value function of alternatives for stakeholders. This value function extends the value function of ExpTODIM proposed by Leoneti and Gomes [33], a multicriteria decision analysis method that has been shown to better approximate the value function of prospect theory [26] as a psychological theory of decision-making under risk that has been shown to have higher predictive capabilities than rational choice theories rooted in expected utility theory [24] and subjective expected utility theory [25].

Definition 2.7 **The value function of alternatives for criterion $c_{j}$ and stakeholder $s_{k}$**

Let $\phi_{j}^{k}$, the value function of alternatives for criterion $c_{j}\in C$ and stakeholder $s_{k}\in S$, with 1≤i,i′≤m, 1≤j≤n, and 1≤k≤o, be defined as a pairwise comparison between each pair of alternatives $a_{i}$ and $a_{i\prime }$ in *A* as follows:(2)
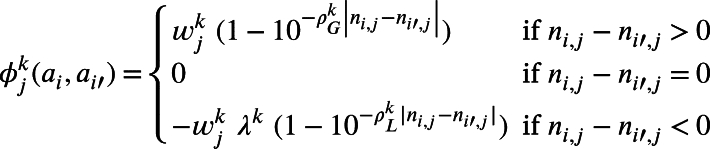
 where $w_{j}^{k}$ is the $j^{th}$ component of the vector ${\overset{\to }{w}}^{k}=(w_{j}^{k})$ used by stakeholder $s_{k}$ to weigh the relevance of criterion $c_{j}$ in *C*, $\rho_{G}^{k}$ and $\rho_{L}^{k}\in N^{⁎}$ are two parameters that moderate the sensibility of stakeholders to gains in the domain of gains and their sensibility to losses in the domain of losses. Higher values of the parameters $\rho_{G}^{k}$ and $\rho_{L}^{k}$ render the value function steeper and thus more sensitive to smaller variations in gains or losses [34]. We also use the factor $\lambda^{k}$, which is a factor used to amplify the losses proposed in prospect theory [26], [35] that renders the convex curve steeper nearer the reference point in the domain of losses and moderate the effect of loss aversion as the most significant psychological effect governing decision-making under risk [26].

Remark 2 We assume the $\lambda^{k}$, the factor of amplification of losses, to range between 1.80 and 2.50 based on cumulative prospect theory [35] and use it to moderate the “losses-loom-larger-than-gains” effect originally proposed in prospect theory [26].

Once the pairwise comparison values haven been calculated using our value function as per Eq. (2), we use the global dominance function to calculate the dominance of alternatives, as per Eq. (3) below.

Definition 2.8 **The dominance function of alternative $a_{i}$ over alternative $a_{i}\prime$ for stakeholders**

Let the dominance of alternative $a_{i}$ over alternative $a_{i\prime }\in A$ for stakeholder $s_{k}\in S$, be defined as follows:

(3) $$\delta_{i}^{k}(a_{i},a_{i\prime })=\sum_{j=1}^{n}\phi_{j}^{k}(a_{i},a_{i\prime })$$

for all 1≤i,i′≤m, 1≤j≤n and 1≤k≤o.

Definition 2.9 **The welfare of alternative $a_{i}$ for stakeholders**

Let the welfare of alternative $a_{i}\in A$ for stakeholder $s_{k}\in S$ be defined as follows:

(4) $$\delta_{i}^{k}=\sum_{i\prime =1}^{m}\delta_{i}^{k}(a_{i},a_{i\prime })$$

for all 1≤i,i′≤m and 1≤k≤o.

### 2.2 Second step: eliciting the social welfare solution of the consortium

Once the welfare of alternatives for stakeholders have been calculated as per Eq. (4), we can elicit the social welfare of alternatives in *A*. Several social welfare functions can be applied to accomplish this second step.

#### 2.2.1 The utilitarian social welfare function

We begin our description of social welfare functions with the utilitarian social welfare function.

Definition 2.10 **The set of social welfares of alternatives**

Let the set of social welfare of alternatives be defined as $U=\{u_{i}\}$, where $u_{i}$, the total welfare of alternative $a_{i}$ across all stakeholders, is given by the following expression:

(5) $$u_{i}=\sum_{k=1}^{o}\delta_{i}^{k}$$

where $\delta_{i}^{k}$ is given by Eq. (4) of Definition 2.9 for all i,k such that 1≤i≤m and 1≤k≤o.

Definition 2.11 **The social welfare solution**

Let the social welfare solution be defined as the supremum of the set *U*.

Remark 3 The social welfare solution introduced by Definition 2.11 is based on the principle of utilitarianism.

#### 2.2.2 The maximax social welfare functions

Although the social welfare function introduced by Definition 2.11 above is a commonly used social welfare function, it is not the only one we can use to propose a social welfare solution for the consortium of stakeholders. Our method can be seamlessly integrated with other social welfare functions as well. In order to show this property of our method, let us introduce other social welfare functions, starting with a social welfare function based on the maximax principle.

Definition 2.12 **The set of maximum welfares of alternatives**

Let the set of maximum welfare of alternatives be defined as $M^{+}=\{max_{i}\}$, where $max_{i}$, the maximum welfare of alternative $a_{i}$ across all stakeholders, is given by the expression:

(6) $$max_{i}=max_{k=1}^{o}(\delta_{i}^{k})$$

where $\delta_{i}^{k}$ is given by Eq. (4) of Definition 2.9, for all i,k such that 1≤i≤m and 1≤k≤o.

Definition 2.13 **The maximax social welfare solution**

Let the maximax social welfare solution be defined as the supremum of the set $M^{+}$.

Remark 4 Definition 2.13 implements the “maximax” principle according to which the alternatives are ranked based on their maximum welfare for stakeholders, which is a more risk-seeking social welfare function based on maximizing welfare.

The maximax social welfare function differs from the utilitarian social welfare function in that the consortium as a whole now adopts a more risk-seeking social welfare function.

#### 2.2.3 The maximin social welfare functions

We now introduce a social welfare function that is the mirror image of the maximax social welfare function and is called the maximin social welfare solution.

Definition 2.14 **The set of minimum welfares of alternatives**

Let the set of minimum welfares of alternatives be defined as $M^{-}=\{min_{i}\}$, where $min_{i}$, the minimum welfare of alternative $a_{i}$ across all stakeholders, is given by the expression:

(7) $$min_{i}=min_{k=1}^{o}(\delta_{i}^{k})$$

where $\delta_{i}^{k}$ is given by Eq. (4), for all i,k such that 1≤i≤m and 1≤k≤o.

Definition 2.15 **The maximin social welfare solution**

Let the maximin social welfare solution be defined as the supremum of the set $M^{-}$.

Remark 5 Definition 2.15 implements the “maximin” principle according to which the alternatives are ranked based on their minimum welfare for stakeholders, which is a more prudent social welfare function based on minimizing losses.

#### 2.2.4 The minimax regrets social welfare functions

Finally, we conclude with a social welfare function based neither on welfares nor on losses but rather on the “regret” perceived by stakeholders.

Definition 2.16 **The set of minimax regrets of alternatives**

Let the set of maximum regrets of alternatives be defined as $R=\{r_{i}\}$, where $r_{i}$, the maximum regret of alternative $a_{i}$ across all stakeholders, is given by the expression:

(8) $$r_{i}=max_{k=1}^{o}(r_{i}^{k})$$

where $r_{i}^{k}$ is given by the expression:

(9) $$r_{i}^{k}=max_{k=1}^{o}(\delta_{i}^{k})-\delta_{i}^{k}$$

where $\delta_{i}^{k}$ is given by Eq. (4), for all i,k such that 1≤i≤m and 1≤k≤o.

Definition 2.17 **The minimax regrets social welfare solution**

Let the minimax regrets social welfare function be defined as the infimum of the set *R*.

## 3 Results

In this section, we present the results obtained using the methodology and the multicriteria group decision analysis method set out in Section 2. We will also compare these results with those obtained with other multicriteria group decision analysis methods and will analyze the main differences.

### 3.1 Applying our method

We will first demonstrate the two-step methodology and present the results obtained using the multicriteria decision analysis methods set out in Section 2 based on a case modeled on the Stratospheric Particle Injection for Climate Engineering (SPICE) project, a climate geoengineering project funded by the Engineering and Physical Sciences Research Council in the UK [36].

#### 3.1.1 Eliciting the welfares of stakeholders (first step)

The first step of our methodology begins with the elicitation of the stakeholders in the consortium.

##### The stakeholders

The stakeholders are a function of the *inclusion* dimension of the RRI construct described in Section 1. We consider as stakeholders the representatives of a research institution, a nongovernmental organization (NGO), and a research-funding agency, which are referred to as $s_{1}$, $s_{2}$ and $s_{3}$, respectively, which we will refer to collective as the consortium. The first step of our methodology continues with the elicitation of alternatives.

##### The alternatives

We consider the alternatives faced by the stakeholders of this project prior to the test-bed deployment of the proposed solution. The alternatives are “proceeding with the test-bed deployment” ($a_{1}$), “backtrack to a previous phase to carry out broader impact assessments” ($a_{2}$), “stop the test-bed deployment in the current phase until more information becomes available” ($a_{3}$), and “cancel the project” ($a_{4}$). These alternatives are modeled on the SPICE project [36, p. 293]. The first step of our methodology continues with the elicitation of criteria.

##### The criteria

We adopt the following criteria under the reflexivity dimension: *compliance* ($c_{1}$), *internal communications* ($c_{2}$), *external communications* ($c_{3}$) and *reviews* ($c_{4}$). The reflexivity criteria are derived from the reflexivity criteria used in the SPICE project, in which the compliance criterion corresponded to “compliance of the test-bed deployment with relevant regulations,” the internal and external communications criteria were subsumed by “clear communication of the nature and purpose of the (SPICE) project,” and the reviews criterion corresponded to “mechanisms to review future applications and impacts” [4, p. 1575]. We also adopt the following criteria under the anticipation dimension: *environmental impact* ($c_{5}$), *social impact* ($c_{6}$), *economic impact* ($c_{7}$), and *political impact* ($c_{8}$).

The first step of our methodology continues with the elicitation of the profiles of stakeholders.

##### The profiles of stakeholders

The profiles of stakeholders are shown in Table 1.

**Table 1:** The profiles of stakeholders.

	*c*_1_	*c*_2_	*c*_3_	*c*_4_	*c*_5_	*c*_6_	*c*_7_	*c*_8_
*r*_1_	3	2	7	8	5	6	1	4
${\overset{\to }{w}}_{1}$	0.1522	0.2147	0.0335	0.0156	0.0793	0.0543	0.3397	0.1106
*r*_2_	6	7	4	5	2	3	8	1
${\overset{\to }{w}}_{2}$	0.0543	0.0335	0.1106	0.0793	0.2147	0.1522	0.0156	0.3397
*r*_3_	3	8	7	6	5	4	2	1
${\overset{\to }{w}}_{3}$	0.1522	0.0156	0.0335	0.0543	0.0793	0.1106	0.2147	0.3397

The results shown in Table 1 were obtained using the ranking order centroid (ROC) method [30]. The ROC method elicits the profiles of stakeholders based on the rankings they assign to criteria, that is, the ROC method takes the rankings $r_{1}$, $r_{2}$, and $r_{3}$ assigned to criteria by each stakeholder and delivers the profiles ${\overset{\to }{w}}_{1}$, ${\overset{\to }{w}}_{2}$, and ${\overset{\to }{w}}_{3}$ of each stakeholder. The first step of our methodology continues with the elicitation of the evaluation matrix of scores.

##### The evaluation matrix of scores

The matrix shown in Table 2 gives us the scores of alternatives [36, p. 293]. This matrix is elicited by all members of the consortium and its values are presented using the Likert scale: very unsatisfactory (1), unsatisfactory (2), neutral (3), satisfactory (4), and very satisfactory (5).

**Table 2:** The matrix of evaluation scores of alternatives.

*E*_*mn*_	*c*_1_	*c*_2_	*c*_3_	*c*_4_	*c*_5_	*c*_6_	*c*_7_	*c*_8_
*a*_1_	1	5	1	2	1	2	5	2
*a*_2_	3	3	2	3	2	4	3	3
*a*_3_	2	4	2	2	3	2	2	3
*a*_4_	4	2	2	1	5	3	1	5

The first step of our methodology ends with the elicitation of the welfares of stakeholders.

##### The welfare of stakeholders

As per Eqs. (1) through Eq. (4) of Definition 2.6 through Definition 2.9, we obtain the welfare of stakeholders shown in Table 3.

**Table 3:** Welfare of alternatives for all stakeholders in the consortium.

	*s*^1^	*s*^2^	*s*^3^
*a*_1_	-1.13581	-5.49212	-3.69256
*a*_2_	-1.04706	-0.50573	-0.36381
*a*_3_	-2.16405	-1.40514	-2.21562
*a*_4_	-2.80185	1.03203	-0.14460

The elicitation of the welfares of stakeholders finalizes the first step of our methodology. The second step of our methodology consists in eliciting the social welfare of the consortium.

#### 3.1.2 Eliciting the social welfare solution of the consortium (second step)

We now show the results of applying the different social welfare functions introduced in Section 2 on the welfares of stakeholders shown in Table 3. We begin our description with one of the most widely used social welfare functions, namely, the utilitarian social welfare function.

##### The utilitarian social welfare solution

As per Eqs. (5) of Definition 2.10 and Definition 2.11, the utilitarian social welfare solution is alternative $a_{4}$, as shown in Table 4.

**Table 4:** Welfare of alternatives and social welfare solution (utilitarian social welfare solution).

	*s*^1^	*s*^2^	*s*^3^	*u*_*i*_	social welfare solution
*a*_1_	-1.13581	-5.49212	-3.69256	-10.3205	
*a*_2_	-1.04706	-0.50573	-0.36381	-1.91660	
*a*_3_	-2.16405	-1.40514	-2.21562	-5.78482	
*a*_4_	-2.80185	1.03203	-0.14460	-1.91442	*a*_4_

Although one of the most widely used social welfare functions, the adoption of a social welfare function based on the utilitarianism principle can lead to undesirable outcomes, such as the well-known “repugnant conclusion effect” of utilitarianism. This effect can arise in situations where, despite having a large number of stakeholders with welfare below a desirable minimum, the total welfare is still maximized by having a small number of stakeholders with a high welfare. Repugnant conclusion brings to our attention the importance of the effects of different welfare functions on social welfare theories [37].

##### The maximax social welfare solution

As per Eq. (6) of Definition 2.12 and Definition 2.13, the maximax social welfare solution is shown in Table 5.

**Table 5:** Welfare of alternatives and social welfare solution (maximax social welfare solution).

	*s*^1^	*s*^2^	*s*^3^	*max*_*i*_	social welfare solution
*a*_1_	-1.13581	-5.49212	-3.69256	-1.13581	
*a*_2_	-1.04706	-0.50573	-0.36381	-0.36381	
*a*_3_	-2.16405	-1.40514	-2.21562	-1.40514	
*a*_4_	-2.80185	1.03203	-0.14460	1.03203	*a*_4_

The maximax social welfare solution takes the utilitarian social welfare function to an extreme level by selecting the alternative that delivers the maximum amount of welfare across all stakeholders in the consortium, which in this case corresponds to alternative $a_{4}$ for the NGO.

##### The maximin social welfare solution

As per Eq. (7) of Definition 2.14 and Definition 2.15, the maximin social welfare solution is shown in Table 6.

**Table 6:** Welfare of alternatives and social welfare solution (maximin social welfare solution).

	*s*^1^	*s*^2^	*s*^3^	*min*_*i*_	social welfare solution
*a*_1_	-1.13581	-5.49212	-3.69256	-5.49212	
*a*_2_	-1.04706	-0.50573	-0.36381	-1.04706	*a*_2_
*a*_3_	-2.16405	-1.40514	-2.21562	-2.21562	
*a*_4_	-2.80185	1.03203	-0.14460	-2.80185	

##### The minimax regrets social welfare solution

As per Eq. (8) and Eq. (9) of Definition 2.16 and Definition 2.17, the minimax regrets social welfare solution is shown in Table 7.

**Table 7:** Welfare of alternatives and social welfare solution (minimax regrets social welfare solution).

	*s*^1^	*s*^2^	*s*^3^	*r*_*i*_	social welfare solution
*a*_1_	0.08875	6.52415	3.54797	6.52415	
*a*_2_	0.00000	1.53776	0.21921	1.53776	*a*_2_
*a*_3_	1.11699	2.43717	2.07102	2.43717	
*a*_4_	1.75479	0.00000	0.00000	1.75478	

The results shown in Table 4 through Table 7 were obtained with the parameters $\rho_{G}^{k}$ and $\rho_{L}^{k}$ set at $\rho_{G}^{1}=\rho_{L}^{1}=5$, $\rho_{G}^{2}=\rho_{L}^{2}=4$ and $\rho_{G}^{3}=\rho_{L}^{3}=1$ and the amplification factor $\lambda^{k}$ set at 2.25 for all stakeholders, that is, for all *k* such that 1≤k≤o. Keeping these values for the parameters $\rho_{G}^{k}$ and $\rho_{L}^{k}$ for all stakeholders, we now set the value of $\lambda^{k}$ for all stakeholders at 2.3. The results are summarized in Table 8.

**Table 8:** Social welfare solutions for all social welfare functions.

Social welfare function	utilitarian	maximax	maximin	minimax regrets
Solution	*a*_2_	*a*_4_	*a*_2_	*a*_2_

This new factor of amplification of losses crosses an upper threshold that prompts the utilitarian social welfare function to switch from alternative $a_{4}$, the preferred solution for the NGO and the research-funding agency, to alternative $a_{2}$, the preferred solution for the research institution. With a value of $\lambda^{k}$ set at 2.3 the maximax social welfare function does not constitute an upper threshold and the value function of the extended ExpTODIM method still delivers alternative $a_{4}$ as the preferred solution and the maximin and minimax regrets social welfare functions render alternative $a_{4}$ even more undesirable. Keeping $\rho_{G}^{1},\rho_{L}^{1},\rho_{G}^{2}$ and $\rho_{L}^{2}$ unaltered, we now set $\rho_{G}^{3}$ and $\rho_{L}^{3}$ at 2 and $\lambda^{k}$ at 1.90 for all stakeholders. The results are shown in Table 9.

**Table 9:** Social welfare solutions for all social welfare functions.

Social welfare function	utilitarian	maximax	maximin	minimax regrets
Solution	*a*_4_	*a*_4_	*a*_2_	*a*_2_

Leaving the parameters $\rho_{G}^{k}$ and $\rho_{L}^{k}$ unaltered, we now set $\lambda^{k}$ at 1.95 for all stakeholders. This factor of amplification of losses crosses an upper threshold that prompts the utilitarian social welfare function to switch from alternative $a_{4}$, the preferred solution for the NGO and the research-funding agency, to alternative $a_{2}$, the preferred solution for the research institution.

The increase in value of the parameters $\rho_{G}^{3}$ and $\rho_{L}^{3}$ from 1 to 2 reduces this threshold from the value of $\lambda^{k}$ = 2.3 to the value 1.95. With $\lambda^{k}$ set at the value 1.95 the maximax social welfare function renders alternative $a_{4}$ as the solution, while the maximin and minimax regrets social welfare functions still render alternative $a_{2}$ as the solution. These results are shown in Table 10.

**Table 10:** Social welfare solutions for all social welfare functions.

Social welfare function	utilitarian	maximax	maximin	minimax regrets
Solution	*a*_2_	*a*_4_	*a*_2_	*a*_2_

### 3.2 Comparison with other methods

Scholarly work on the integration of prospect theory with multicriteria decision analysis has so far focused on the development of psychologically grounded multicriteria analysis methods for individual decision-making. As mentioned in Section 2, TODIM was proposed in 1991 as the first psychologically grounded multicriteria analysis method for individual decision-making [38]. Since then, several psychologically grounded multicriteria decision analysis methods based on TODIM have been proposed, including the extended ExpTODIM we introduced in Section 2.

The most notorious case of a psychologically grounded multicriteria analysis method for individual decision-making not based on TODIM is the behavioral TOPSIS method introduced by Yoon and Kim [39] in 2017. Behavioral TOPSIS is based on TOPSIS, a multicriteria analysis method for individual decision-making that was introduced in 1981 and has become one of the most widely used multicriteria analysis method for individual decision-making due to the computational tractability of its value function and its predictive capability [40].

While several multicriteria methods for group decision-making have been proposed in the multicriteria decision analysis literature, scholarly work on the integration of prospect theory with these methods has limited itself to the application of existing multicriteria group decision analysis methods, such as the TOPSIS for groups method, in niche domains that require the deployment a fuzzy logic [41]. To compare our method with a psychologically grounded multicriteria analysis method for group decision-making we will use the TOPSIS for groups method [42], a widely used multicriteria analysis method for group decision-making, in conjunction with the behavioral TOPSIS method [39].

#### 3.2.1 Applying the TOPSIS for groups method

The TOPSIS for groups method is based on the TOPSIS method introduced by Hwang and Yoon [40]. The value function of the original TOPSIS method is based on a metric of closeness to the ideal solution that is calculated based on the distance to the positive ideal solution (DPIS) and the distance to the negative ideal solution (DNIS). The idea behind the TOPSIS method is that a solution is preferred to the extent that it is closer to the ideal positive solution and farther away from the negative ideal solution.

Following the same logic, the TOPSIS for groups method introduced by Shih, Shyur, and Lee [42] in 2007 aggregates the distances to the positive and negative ideal solutions of each stakeholder into a cumulative distance to the positive ideal solution (CDPIS) and a cumulative distance to the negative ideal solution (CDNIS) using a multiplicative function. This function uses the distance to the positive ideal solution and the distance to the negative ideal solution calculated using the original TOPSIS method [40] for each stakeholder. TOPSIS for groups then calculates the “relative closeness to the ideal solution” (RCIS) with the formula RCIS=CDNIS/(CDPIS+CDNIS). Using this index of closeness, the solution for the group of stakeholders is the alternative with the highest closeness to the ideal solution. As shown in Table 11, alternative $a_{4}$ yields the highest value of closeness and is thus the preferred solution.

**Table 11:** Welfare of alternatives (calculated using TOPSIS for groups).

	*CDPIS*	*CDNIS*	*RCIS*	ranking	solution
*a*_1_	0.39085	0.25431	0.39418	3	
*a*_2_	0.28464	0.27216	0.48879	2	
*a*_3_	0.33226	0.20145	0.37744	4	
*a*_4_	0.29469	0.37591	0.56056	1	*a*_4_

#### 3.2.2 Applying the TOPSIS for groups with the behavioral TOPSIS method

In this section, we compare our method with one of the most cited and widely used multicriteria methods for group decision-making not based on TODIM [38], namely, the TOPSIS for group method [39], in conjunction with the behavioral TOPSIS method, a well-known multicriteria decision analysis method for individual decision-making introduced in 2017 that integrates in its value function the factor of amplification of losses *λ* of prospect theory, as originally proposed in [26] and further developed in [35].

When used in conjunction with TOPSIS for groups, the value function of behavioral TOPSIS amplifies the distance to the cumulative positive ideal solution as a proxy of losses of stakeholders by the factor of amplification of losses *λ* proposed in prospect theory [26]. This is accomplished by replacing the relative closeness to the ideal solution (RCIS) of TOPSIS for groups with the value function V=CDNIS−λ×CDPIS, where the amplification factor $\lambda^{k}$ of the extended ExpTODIM value function used by our method is replaced with *λ*, the amplification factor used by behavioral TOPSIS, which is assumed to be equal in value for all stakeholders. The end result is a metric of distance *V* according to which an alternative $a_{i}$ is preferred over an alternative $a_{i\prime }$ to the extent that $a_{i}$ yields a higher value of *V* than $a_{i\prime }$.

Applying behavioral TOPSIS in conjunction with TOPSIS for groups, and setting the value of $\lambda^{k}$ for all stakeholders at $\lambda^{k}=2.25$, alternative $a_{4}$ yields the highest value of closeness and is thus the preferred solution, as shown in Table 12.

**Table 12:** Welfare of alternatives (calculated using TOPSIS for groups with behavioral TOPSIS).

	*CDPIS*	*CDNIS*	*V*	ranking	solution
*a*_1_	0.39085	0.25431	-0.6251	4	
*a*_2_	0.28464	0.27216	-0.3683	2	
*a*_3_	0.33226	0.20145	-0.5462	3	
*a*_4_	0.29469	0.37591	-0.2871	1	*a*_4_

### 3.3 Analysis of results

Although TOPSIS treats the distances DPIS and DNIS as proxies of gains and losses, respectively, these metrics of distance do not correlate with the actual gains and losses to a reference point described in prospect theory [26]. This leads to a loss of information that makes it difficult to integrate the value function of prospect theory in the TOPSIS method. Moreover, the calculation of the index RCIS based on the cumulative distances uses a multiplicative instead of an additive function. This results in a further loss of information that turns the original TOPSIS method [40] less appropriate to implement a psychologically grounded multicriteria analysis method for individual and group decision-making based on prospect theory.

This overall loss of information leads to a number of potential problems, such as the cancellation effect that may occur during the aggregation of individual preferences of stakeholders. By calculating the relative distance to the ideal positive and negative alternatives after aggregation instead of actual gains and losses relative to a reference point, the TOPSIS for groups method does not ensure that the preferences and evaluations of each stakeholder are preserved and reflected in the proposed solution.

Besides being a first psychologically grounded multicriteria decision analysis method for group decision-making, our method constitutes an advancement over TOPSIS for groups used in conjunction with behavioral TOPSIS by more effectively capturing the essence of loss aversion as the main psychological effect in individual and group decision-making under risk. The reason for this lies in the extended ExpTODIM value function used by our method, which is able to model different levels of sensibility to variations in gains and losses as a direct result of integrating in its value function the parameters $\rho_{G}^{k}$ and $\rho_{L}^{k}$ and the amplification factor $\lambda^{k}$. Unlike behavioral TOPSIS, which was developed based on TOPSIS only as an afterthought, the extended ExpTODIM value function proposed in Section 2 has been designed to better approximate the value function of prospect theory from the outset.

While under some configurations of the sensibility parameters and the factor of amplification of losses our results are consistent with the results proposed by the two methods we have chosen for comparative purposes, we also show how the proposed solutions change as a function of the values of the sensibility parameters and the factor of amplification of losses. This is a fundamental difference, as TOPSIS for groups, when used in conjunction with behavioral TOPSIS to model the psychology of individual decision-making in the domain of losses, regards the amplification factor as a property of the value function that remains constant for all stakeholders, and not as a property of the stakeholders.

The sensibility parameters introduced in the value function of our method also allow us to model varying degrees of sensibility to changes in gains and losses on the part of stakeholders, which has an impact on the degree of risk aversion of stakeholders in the domain of gains and on the risk propensity in the domain of losses and may lead to different results. We demonstrate this by eliciting thresholds of robustness for the solutions proposed based on different value configurations for the parameters and the factor of amplification used in the value function of our method.

## 4 Discussion

We begin this section with a discussion of the contributions of our method.

### 4.1 Contributions of the method

In this article, we contribute to extant literature in the emerging field of RRI by setting out a formal decision-theoretic method that construes the governance of RRI as a social welfare problem. Our method can be used to develop decision-aiding tools allowing research and innovation managers at universities, contract research institutions, large diversified firms, and small and medium-sized enterprises to account for any negative externalities of their research and innovation agendas, whether anticipated or not, and to respond to internal and external stakeholders and to society at large. Following a multicriteria group decision analysis approach, our method informs the complex trade-offs that very often arise when driving research and innovation in large consortia involving stakeholders from industry and academia.

Our method follows an evolutionary approach to the governance of RRI whereby the decisions made by internal and external stakeholders flow dynamically as an innovation project unfolds through the innovation lifecycle over time. Due to their strategic importance, our method accommodates the inclusion of external stakeholders that often disembark uninvited in a research and innovation project, such as nongovernmental organizations (NGOs) and civil society organizations (CSOs). These often uninvited external stakeholders are a special class of stakeholders that influence the decisions of incumbent stakeholders in the innovation ecosystem of an innovation project in a very conspicuous way, often holding them accountable for any negative externalities and forcing them to respond to society [14], [43].

Our method also contributes to extant literature in the field of RRI by setting a novel research agenda at the intersection of the emerging field of RRI, on the one hand, and the more mature fields of multicriteria decision analysis [44] and welfare economics and social choice theory [28], on the other. Such an integration holds great promise, as the governance of RRI can greatly benefit from integrating psychologically grounded approaches to individual decision-making under uncertainty rooted in multicriteria decision analysis and behavioral economics as well as group decision and negotiation approaches to collective decision-making rooted in welfare economics and social choice theory [29], [45], [46].

Our method can seamlessly integrate social welfare functions that are well known and have a long tradition in the social choice theory and welfare economics literatures [27], which can contribute to informing collective decisions pertaining to RRI in projects that are likely to raise RRI issues in complex and rapidly evolving innovation ecosystems comprised of a large number of external stakeholders, including NGOs, CSOs and transnational government institutions, to name but a few. [47].

### 4.2 Comparison with previous approaches

The comprehensive review of RRI literature conducted by Stilgoe, Owen, and Machnaghten [4, p. 1576] concluded that “the case [of the SPICE project] highlights the potential for a framework to inform decision-making in a field with limited governance” [4]. In what constitutes a first systematic review of research on the development and use of monitoring and evaluation tools for RRI since 2013, Monsonís-Payá, Iñigo, and Blok [16] revealed that a diverse pool of 25 different tools is commonly used to monitor and evaluate RRI not only in connection with research and innovation projects, strategies and policies but also for assessing the RRI readiness of organizations engaged in research and innovation [16, p. 11].

While the use of taxonomies of criteria and indicators for implementing evaluation and assessment methodologies is common to most of the tools analyzed by these authors, the use of formal decision-theoretic tools in general, and those based on multicriteria decision analysis in particular, remains underrepresented. Notable exceptions elicited by these authors include cases reporting the use of the Analytical Hierarchy Process [31] for evaluating RRI policies, the use of qualitative multicriteria self-questionnaires for self-assessment, and the use of stage-gating methodologies [48] involving social criteria for evaluating the RRI readiness of firms.

This study focused on articles published in the Journal of Responsible Innovation as the leading journal in this field [16, p. 5]. Its results show that multi-stakeholder, multicriteria decision analysis methods rooted in decision sciences, with its potential to implement decision-aiding tools *in silico*, are yet to be more broadly adopted for the governance of RRI, as suggested by Stilgoe, Owen, and Macnaghten [4, p. 1576], to implement the dimension of responsiveness ten years ago. This systematic review reveals that little progress has been made along these lines in the last decade and brings to our attention the big gap between the adoption of decision-theoretic methods and tools in the field of RRI, as “a field with limited governance” [4, p. 1576], when compared with other areas in the management sciences [44]. Our method contributes to bridging this gap in the RRI literature.

Our selection of the value function of ExpTODIM in Section 2 was based on the results of the field study and comparative analysis that Leoneti and Gomes [33] conducted between the value function of ExpTODIM and other psychologically grounded value functions proposed in the multicriteria decision analysis literature, including the value function of the original TODIM method [38] and its revision [49], and those of its various extensions [50], [51], [52], [53], [54].

We have extended the value function of ExpTODIM as per Eq. (2) in Section 2 in order to propose a psychologically grounded method for the governance of RRI following a social welfare approach. In so doing, we have also delivered a novel application in the field of multicriteria decision analysis [44].

### 4.3 Toward psychologically grounded methods for the governance of RRI

Our method follows a two-step process that consists in eliciting the welfare of stakeholders and aggregating them to propose the social welfare of a research and innovation consortium.

#### 4.3.1 Computing the welfare of stakeholders

In a first step, our method computes the welfares of stakeholders not using an ordinal preference but rather a cardinal measurement of welfare. This measurement of welfare conveys more information than ordinal preferences, allows for intra- and interpersonal comparisons, and is calculated using a value function that approximates the value function of prospect theory [35]. The value function introduced by Eq. (2) in Section 2 extends the value function of ExpTODIM [33] to capture the effect of loss aversion as the main psychological effect governing human-decision making under risk [45], [46] more accurately than other psychologically inspired multicriteria decision analysis methods [33], [38], [49], [50], [52], [53], [54].

Our extensions to the value function of ExpTODIM allow us to expand the profiles of stakeholders, which were initially modeled only in terms of the weights attached to criteria, with information allowing us to model the risk aversion of stakeholders in the domain of gains, their propensity to risk in the domain of losses, and the amplification of losses near the reference point in the domain of losses. This is accomplished through the sensibility parameters $\rho_{G}^{k}$ and $\rho_{L}^{k}$ and through the amplification parameter $\lambda^{k}$ introduced by Eq. (2) of Definition 2.7 in Section 2. These parameters and the amplification factor can be set at different values to moderate the psychological effects of loss aversion as part of the profiles of stakeholders, which allows us to mimic the psychology of human decision-making under uncertainty and renders the value function of Eq. (2) more psychologically plausible.

#### 4.3.2 Computing the social welfare of the consortium

In a second step, the measurement of welfare of stakeholders is aggregated to calculate the total social welfare. This process can be achieved using different social welfare functions, which will produce different results depending on the principles of social welfare they are based upon. We can attribute a behavioral classification to a consortium by virtue of it endorsing a given social welfare function, such as a risk-seeking behavior to a consortium endorsing the maximax social welfare function with its focus on maximizing welfare, or a risk-averse behavior to another consortium endorsing the maximin social welfare function with its focus on minimizing losses. However, the lack of psychologically grounded theories of collective decision-making makes the integration of the social psychology of group decision-making under uncertainty much more challenging [45], [46].

#### 4.3.3 The advantages of psychologically motivated methods for RRI governance

The deployment of psychologically motivated multicriteria group decision analysis methods for RRI governance has a number of advantages. These methods show greater predictive capabilities than those that follow conventional rational choice approaches based on expected utility theory [24] and subjective expected utility theory [25]. They allow us to implement decision-support and decision-augmented systems *in silico* with lower levels of algorithm aversion, especially when these systems are used as decision-theoretic tools for assessing social welfare [55].

Psychologically motivated multicriteria group decision analysis methods for RRI governance also allow us to implement agent-based models *in silico* for social simulation and policy engineering in the area of research and innovation that mimic the way humans go about making complex decisions under risk based on multiple and often conflicting criteria. This leads to social simulations that have higher predictive capabilities as they implement more realistic agent-based models for policy engineering [20] following a psychologically grounded [45] and critical realist approach [56].

#### 4.3.4 NGOs and CSOs and mechanisms of RRI governance

Nongovernmental organizations (NGOs) and civil society organizations (CSOs) have emerged as mechanisms of RRI governance and their influence as such is expected to increase in the future [14], [43]. This begs the question of how to manage conflicts between them and the usual stakeholders that are part of RRI consortia and how to ensure their meaningful participation.

NGOs, and especially CSOs, are a very special kind of stakeholder. Although they may in some cases be part of the research and innovation consortia from the outset, they often become, and are treated as, uninvited stakeholders. This precludes their meaningful participation as new stakeholders with the right to participate in the individual and group decision-making process of large consortia. To the extent that they become part of the set *S* of stakeholders, they can more meaningfully participate as cocreators in research and innovation projects, which would correspond to the highest level of the dimension inclusion of the RRI construct shown in Fig. 1. Lower levels of the dimension inclusion would lead to governance models such as the consultative model, in which case they would only participate in a consultative capacity without any voting rights [14].

However, conflict resolution may arise not only with NGOs and CSOs but also with any other stakeholder in general. To the extent that the stakeholders in *S* can agree on an initial set of criteria *C* above a threshold of relevance, conflicts will arise so long as they assign substantially different weights to the set of criteria in *C*. How to resolve these conflicts is a question that is addressed in the second step or our methodology through a suitable social welfare function that the consortium adheres to in order to come up with a group solution. Our method is modular enough to accommodate any number of social welfare functions. So far, we have included four different social welfare functions. But the potential to develop and integrate a large number of other social welfare functions is very high.

#### 4.3.5 Beyond the governance of RRI

The problem of conflict resolution among heterogeneous stakeholders using multiple criteria is not exclusive to the governance of RRI. Consider for example the difference in terms of governance model between the stock corporation, which reduces the set *S* of stakeholders with voting rights to include only shareholders, allowing each one of them not only to be part of the set *S* by virtue of acquiring and holding shares of the corporation but also to hold any number of shares, and the governance model of a consumer cooperative, which allows only consumers with a vested interest in receiving goods and services from the cooperative to be elements of the set *S*.

While in the stock corporation conflicts are resolved by applying the principle of one share one vote, thus allowing majority shareholders to control the corporation in a straightforward manner, conflict resolution in cooperatives is generally more complex because they adhere to one the main cooperative principles that governs cooperative decision-making, namely, the principle of one member one vote, which precludes a reduced number of members to control the cooperative.

While most decisions in the stock corporation are dealt with by the executive team, cooperatives tend to include all stakeholders in strategic decision-making. This is one of the reasons why multicriteria decision analysis approaches have already been proposed to model the governance of cooperative firms [57]. Despite these initial developments, the use of multicriteria decision analysis methods for the governance of stock corporations, cooperatives, and other legal forms, and also for the governance of RRI, is still marginal today [16], [21].

The governance of RRI is one the most complex forms of governance for a number of reasons. Large projects of research and innovation often take place in large consortia comprised of loosely coupled stakeholders with often conflicting objectives. Technological innovation is also ridden with ethical trade-offs that are difficult to resolve and can render research and innovation projects highly RRI-sensitive. The fact that innovation is inherently uncertain also makes individual and collective decision-making subject to the biases described by prospect theory. For all these reasons, the development of governance models of RRI based on multicriteria decision analysis can spearhead not only the governance of RRI but also the governance in other areas of the social sciences.

### 4.4 Toward a general framework for the governance of RRI

Our method is modular enough to be extended and become a general framework for the governance of RRI.

#### 4.4.1 Generalizing our method

Future empirical validation may result in extending the construct with additional dimensions and subdimensions. This would require the instantiation of additional criteria as elements of the set *C*, which is a process that can be accommodated straightforwardly in our method. Adding additional stakeholders as part of the delineation of the innovation ecosystem of a project, which are a result of changes in the inclusion dimension, can also be accommodated straightforwardly by adding elements to the set of stakeholders *S*. The profiles of stakeholders, which are modeled using the weights each stakeholder attaches to each criterion [30], [31], [58], can also be modified by eliciting a new vector ${\overset{\to }{w}}^{k}$.

Psychological effects of human decision-making under risk, which are captured through the sensibility parameters $\rho_{G}^{k}$ and $\rho_{L}^{k}$ and the amplification factor $\lambda^{k}$, can also be estimated and instantiated in our model and can contribute to expanding the decision-making profiles of stakeholders to make them more psychologically grounded [35]. Changes in some of the assumptions of our model can also be accommodated straightforwardly, such as the social welfare principle used to implement the social welfare function required in the second step of our method.

In order to become a general framework for the governance of RRI, however, our method needs to be extended in several areas. We elaborate on some of the needed extensions below.

#### 4.4.2 Limitations and suggestions for future research directions

Several challenges remain open and will need to be addressed by future work toward the ultimate goal of proposing a general framework for the governance of RRI. They include the question of how the interplay between the decisions of stakeholders in the first step and the decisions of the consortium in the second step of our method can induce changes in the social welfare function applied beyond those presented in this article. Indeed, our discussion of social welfare functions has only scratched the surface of a problem that is not only complex but also of paramount importance for the governance of RRI as a social process of building social welfare in society.

Methods for eliciting the parameters and the amplification factor of the value function used by our method beyond those used and applied by Tversky and Kahneman [35] shall also be incorporated in order to provide more psychologically grounded value functions. We have so far relied on the values estimated by these authors for the factor of amplification of losses [35, p. 311] but future interdisciplinary work at the intersection of psychology and decision sciences is needed to render the profiles of stakeholders more psychologically grounded when making decisions about perceived gains, losses, and regrets under uncertainty.

Another aspect that needs to be incorporated in our method deals with the dynamics of decision-making under risk. Changes in the context of a project, such as changes in the dimension of inclusion inducing the adoption of new stakeholders, may lead to changes in the social welfare function used, for example through the introduction of veto powers vested on some of the new stakeholders, which may alter the decision-making powers of stakeholders. Or they may induce changes in the weights of stakeholders leading to changes in individual preferences. The case of NGOs and CSOs is especially relevant here, as these types of stakeholders can parachute as uninvited stakeholders into a project and may lead to the adoption of social welfare functions based on a principle of “prioritarianism,” which would regard the amount of social welfare that accrues to a large group of stakeholders in society, for example those represented by NGOs or CSOs, as having much higher priority.

When endowed with the sense of urgency, legitimacy, and the power required to install inducement mechanisms in a research and innovation consortium, external stakeholders such as NGOs and CSOs can quickly become a mechanism of governance of RRI and change the evolutionary path of research and innovation projects, especially those that are regarded as RRI-sensitive by CSOs. As they can surge suddenly and unexpectedly and gain legitimacy and power quickly, CSOs can have a major impact on the governance of RRI-sensitive projects.

In the case of the SPICE project, NGOs were present from the beginning. Unfortunately, cases such as the SPICE project are not often disclosed, let alone comprehensively analyzed and discussed in detail in the RRI literature [4]. The thorough discussion of RRI-sensitive projects such as the SPICE project is rather exceptional. This situation limits our ability to understand not only how NGOs and CSOs engage with research and innovation consortia in industry and academia but also the nature of the mechanisms by which they can influence the decision-making processes of stakeholders in RRI-sensitive projects. As the governance of RRI adopts more inclusive models of RRI governance, such as the consultative and co-constructive models [59], [60], we expect that the gradual inclusion of NGOs and CSOs, whether in a consultative or co-creative capacity, will mitigate this problem.

## 5 Conclusions

The method set out in this article extends that state of the art in the development of mechanisms and tools for the governance of RRI, as described by Monsonís-Payá, Iñigo, and Blok [16], and extends extant RRI literature by proposing multicriteria group decision analysis methods following a novel social welfare approach. There is an important postulate behind this new approach, namely, that RRI is predicated on the aggregated social welfare that new technological innovations can introduce in society. This postulate leads to a new definition of RRI that goes beyond those described thus far in the RRI literature. In our view, this “RRI-as-social-welfare” definition has a number of important advantages.

By construing RRI as social welfare, this definition allows us to deploy a measurable and quantifiable variable, as opposed to the variable of “responsiveness” in the definition offered by Stilgoe, Owen, and Macnaghten [4] and the variables of “acceptability,” “sustainability,” and “societal desirability” in the definition offered by von Schomberg [1], [2], [3], all of which remain qualitative in nature. Defined in terms of social welfare, RRI, as a novel research field, also benefits from the comprehensive body of work in social choice theory and welfare economics [61]. This allows us to integrate a wide range of social welfare principles, rules, norms, and functions as part of the second step of our method in a rather straightforward manner.

Our method draws upon a model that construes the governance of RRI as a multi-stakeholder, multicriteria group decision analysis problem of delivering social welfare in society and contributes to set a research agenda at the intersection of RRI, multicriteria group decision analysis, social choice theory, and welfare economics. This research agenda contributes to advancing the field of RRI by proposing methods for RRI governance as a process of delivering social welfare in society that takes place in complex innovation ecosystems comprised of heterogeneous stakeholders. This research agenda has not only theoretical but also practical implications. Our method can indeed be used to develop decision-theoretic tools that can be used by practitioners and policymakers alike to resolve the complex trade-offs arising in connection with the governance of RRI based on principles of social welfare.

Further investigation of the role that reciprocal interactions among heterogeneous stakeholders play in the governance of RRI is also needed. As they can greatly influence the governance decisions of innovation consortia, reciprocal interactions and their interplay with loss aversion as a key psychological effect governing individual decision-making under risk will need to be formally described and integrated in our method. The effects of group size on reciprocity and other social interactions within groups, as suggested by work on the effect of reciprocity on strategic thinking in games [45] and behavioral economics [46], will also need to be formally described and incorporated in the individual welfare and social welfare functions of our method.

## Ethics statement

The authors declare that the article is/was not under consideration for publication elsewhere, that the work described in this article has not been published previously, that the authors have made substantial contributions to the conception and design of the study, or acquisition of data, or analysis and interpretation of data, to the drafting of the article or revising it critically for important intellectual content, and to the final approval of the version to be submitted, that the article's publication has been approved by all authors and tacitly or explicitly by the responsible authorities where the work was carried out, and that the article will not be published elsewhere in the same form, including electronically, without the written consent of the copyright-holder. The corresponding author was supported by a grant from the 10.13039/100011102European Commission Seventh Framework Programme for Research and Technological Development under grant number 321480 and the authors declare that the research was conducted in the absence of any commercial or financial relationships that could be construed as a potential conflict of interest.

## CRediT authorship contribution statement

**Harold Paredes-Frigolett:** Writing – review & editing, Writing – original draft, Investigation, Formal analysis, Data curation, Conceptualization. **Andreas Pyka:** Writing – review & editing, Supervision, Methodology, Funding acquisition, Formal analysis, Conceptualization. **Alexandre Bevilacqua Leoneti:** Validation, Data curation, Conceptualization. **Pablo Nachar-Calderón:** Validation, Formal analysis, Conceptualization.

## Declaration of Competing Interest

The authors declare that they have no known competing financial interests or personal relationships that could have appeared to influence the work reported in this paper.

## Data Availability

All data sources used have been cited throughout this article and can be accessed using the references provided.
